# Discovery of FOCAD: An Important Gene in Liver Cirrhosis

**DOI:** 10.1055/s-0042-1758351

**Published:** 2022-12-08

**Authors:** Jinjin Shao

**Affiliations:** 1 Center of Safety Evaluation and Research, Hangzhou Medical College, Hangzhou, Zhejiang, People's Republic of China; 2 Key Laboratory of Drug Safety Evaluation and Research of Zhejiang Province, Hangzhou Medical College, Hangzhou, Zhejiang, China

Liver cirrhosis is the 11th most common cause of death, causing more than 1 million deaths globally each year, and together with liver cancer, it accounts for 3.5% of all deaths worldwide. 1 Cirrhosis develops due to long-term chronic liver inflammation, with the replacement of healthy liver parenchyma with diffuse liver fibrosis and regenerative nodules, leading to portal hypertension and various complications and even hepatocellular carcinoma. 2 3 4 The management of cirrhosis is mainly based on the treatment of cause and complications including comprehensive supportive care. However, there are currently no effective drug therapies to cure the disease, and liver transplantation remains the gold standard treatment for cirrhosis. 5 6 Given that cirrhosis has brought heavy health and economic burden to many countries, there is an urgent need for in-depth study of the pathogenesis and key factors of cirrhosis to find feasible intervention strategies.

Cirrhosis is traditionally considered a late-onset disease that appears in adults following environmental factors, such as viral infection, a high-fat diet, or chronic alcoholism. 7 8 Thus, cirrhosis caused by genetic factors appears to have received less attention than environmental factors, and the etiology in infants and young children is far less understood. In a recent study published in *Nature Genetics* , titled “Loss of FOCAD, manipulated through the SKI messenger RNA surveillance pathway, leads to a pediatric syndrome with cirrhosis,” Traspas and colleagues uncovered the essentiality of the FOCAD gene in maintaining liver health and provided evidence that loss-of-function mutations in FOCAD may contribute to cirrhosis in children. 9

The authors reported 14 children from 10 unrelated families in seven countries presenting with a multisystem syndrome characterized by severe neonatal cirrhosis. By combing genome/exome sequencing, a novel animal model of the human disease, and in vitro biological systems, the team identify that the FOCAD gene is indispensable for maintaining human liver health. Mutations in this gene cause a form of early-onset cirrhosis that has not been documented before. Using CRISPR-Cas9 technology, they established in vitro and in vivo FOCAD knockout models to further study the cellular and molecular mechanisms of pediatric cirrhosis. Phenotypic replication of human disease in FOCAD-deficient zebrafish reveals features of altered messenger RNA degradation processes in the liver. FOCAD deficiency in patient primary cells and human liver cell lines impairs the SKI mRNA surveillance pathway by reducing levels of the RNA helicase SKIC2 and its cofactor SKIC3. Compared with other cell types, hepatocytes rely heavily on this mechanism. Hepatocytes exhibited a decrease in albumin expression and overproduction of the cytokine CCL2 following FOCAD knockout, which may play a key role in the progression of cirrhosis. These findings reveal the importance of FOCAD in maintaining liver homeostasis and the potential for therapeutic intervention by inhibiting the CCL2/CCR2 signaling axis.

There are few reports on the biological function of FOCAD, mainly involving focal adhesions, microtubule dynamics, cell cycle regulation, and cancer susceptibility. 10 11 The authors found the novel function that the presence of FOCAD is critical for maintaining the protein half-life of SKIC2 and SKIC3, two important components of the super-killer (SKI) complex. The SKI complex is a cytoplasmic cofactor and regulator of RNA-degrading exosomes. In human cells, the SKI complex functions primarily in the cotranslational surveillance decay pathway, but the mechanism by which damaged SKI complex destroys hepatocytes remains unclear. RNA degradation is involved in the processing, turnover, and monitoring of almost all RNAs in eukaryotic cells and is therefore a central process in gene expression. The authors speculate that certain transcripts that are normally degraded by the SKI mRNA monitoring pathway may accumulate and cause specific toxicity to hepatocytes. One example is the cytokine CCL2, which attracts immune cells and promotes liver inflammation, possibly a key driver of disease progression.

Monocyte chemoattractant protein 1 (MCP-1, CCL2) is the major ligand for the chemokine receptor C-C chemokine receptor 2 (CCR2) and is involved in a variety of diseases, including autoimmune diseases, viral infections, and cancer. During liver injury, CCL2 and CCR2 promote liver fibrosis by activating inflammatory signaling and immune cell infiltration. CCL2 is a potential therapeutic intervention point for patients with cirrhosis. 12 13 14 At present, drug development targeting this pathway is in full swing, and most of the research has focused on small molecules and monoclonal antibodies targeting CCR2. Cenicriviroc (CVC) is a first-in-class oral dual CCR2/CCR5 antagonist developed by Tobira Therapeutics. CVC has potent anti-inflammatory and antifibrotic activities and is currently in clinical development for the treatment of liver fibrosis. 12 15 According to the results of its 2-year Phase 2b clinical study (CENTAUR study), CVC is well tolerated and has antifibrotic activity in adult patients with nonalcoholic steatohepatitis and fibrosis. 16 The present study further showed the overproduction of CCL2 in FOCAD-deficient patients and provided novel theoretical support for targeting the CCL2–CCR2 signaling axis as a potential target for liver disease patients.

Overall, this study confirms the clinical impact of recessive loss-of-function variants in the FOCAD gene and highlights the importance of the SKI mRNA surveillance pathway for liver homeostasis, and discloses a possible therapeutic intervention point via inhibition of the CCL2/CCR2 signaling axis. Although this study aimed to identify the underlying genetic causes of hepatic syndrome in children, it also has profound implications for the exploration of the pathogenesis of cirrhosis in adults. The relationship between abnormal FOCAD function and the susceptibility and severity of cirrhosis in adults remains to be further explored. In addition, further analysis of cirrhosis cases in different environmental backgrounds is promising to elucidate the genetic component of cirrhosis and its dependence on the environment. Furthermore, the knowledge and tools generated from this study have the potential to help discover and validate innovative targets for other common liver diseases such as fatty liver and hepatocellular carcinoma.

## References

[1] Ginès P Krag A Abraldes J G Solà E Fabrellas N Kamath P S Liver cirrhosis Lancet 2021 398(10308):1359 1376 34543610 10.1016/S0140-6736(21)01374-X

[2] Engelmann C Clària J Szabo G Bosch J Bernardi M Pathophysiology of decompensated cirrhosis: portal hypertension, circulatory dysfunction, inflammation, metabolism and mitochondrial dysfunction J Hepatol 2021 75 01 S49 S66 34039492 10.1016/j.jhep.2021.01.002 PMC9272511

[3] Garrido A Djouder N Cirrhosis: a questioned risk factor for hepatocellular carcinoma Trends Cancer 2021 7 01 29 36 32917550 10.1016/j.trecan.2020.08.005

[4] Baba M R Buch S A Revisiting cancer cachexia: pathogenesis, diagnosis, and current treatment approaches Asia Pac J Oncol Nurs 2021 8 05 508 518 34527780 10.4103/apjon.apjon-2126 PMC8420916

[5] Caraceni P Abraldes J G Ginès P Newsome P N Sarin S K The search for disease-modifying agents in decompensated cirrhosis: from drug repurposing to drug discovery J Hepatol 2021 75 01 S118 S134 34039483 10.1016/j.jhep.2021.01.024

[6] Lim C Turco C Balci D Auxiliary liver transplantation for cirrhosis: from APOLT to RAPID: a scoping review Ann Surg 2022 275 03 551 559 34913893 10.1097/SLA.0000000000005336

[7] Sheka A C Adeyi O Thompson J Hameed B Crawford P A Ikramuddin S Nonalcoholic steatohepatitis: a review JAMA 2020 323 12 1175 1183 32207804 10.1001/jama.2020.2298

[8] Peng C Y Chien R N Liaw Y F Hepatitis B virus-related decompensated liver cirrhosis: benefits of antiviral therapy J Hepatol 2012 57 02 442 450 22504333 10.1016/j.jhep.2012.02.033

[9] Traspas R M Teoh T S Wong P M Loss of FOCAD, operating via the SKI messenger RNA surveillance pathway, causes a pediatric syndrome with liver cirrhosis Nat Genet 2022 54 08 1214 1226 35864190 10.1038/s41588-022-01120-0 PMC7615854

[10] Brand F Förster A Christians A FOCAD loss impacts microtubule assembly, G2/M progression and patient survival in astrocytic gliomas Acta Neuropathol 2020 139 01 175 192 31473790 10.1007/s00401-019-02067-z

[11] Brockschmidt A Trost D Peterziel H KIAA1797/FOCAD encodes a novel focal adhesion protein with tumour suppressor function in gliomas Brain 2012 135(Pt 4):1027 1041 22427331 10.1093/brain/aws045

[12] Ambade A Lowe P Kodys K Pharmacological inhibition of CCR2/5 signaling prevents and reverses alcohol-induced liver damage, steatosis, and inflammation in mice Hepatology 2019 69 03 1105 1121 30179264 10.1002/hep.30249 PMC6393202

[13] Lan T Li C Yang G Sphingosine kinase 1 promotes liver fibrosis by preventing miR-19b-3p-mediated inhibition of CCR2 Hepatology 2018 68 03 1070 1086 29572892 10.1002/hep.29885 PMC6174945

[14] Diao F Single-cell landscape of liver cancer in response to immunotherapy Asia Pac J Oncol Nurs 2021 8 06 591 593 34790841 10.4103/apjon.apjon-2165 PMC8522590

[15] Tacke F Cenicriviroc for the treatment of non-alcoholic steatohepatitis and liver fibrosis Expert Opin Investig Drugs 2018 27 03 301 311 10.1080/13543784.2018.1442436 29448843

[16] Ratziu V Sanyal A Harrison S A Cenicriviroc treatment for adults with nonalcoholic steatohepatitis and fibrosis: final analysis of the phase 2b CENTAUR study Hepatology 2020 72 03 892 905 31943293 10.1002/hep.31108

